# Impaired Sensorimotor Integration in Restless Legs Syndrome

**DOI:** 10.3389/fneur.2018.00568

**Published:** 2018-07-11

**Authors:** Yicong Lin, Yijin Wang, Shuqin Zhan, Yan Ding, Yue Hou, Li Wang, Yuping Wang

**Affiliations:** ^1^Department of Neurology, Xuanwu Hospital, Capital Medical University, Beijing, China; ^2^Beijing Key Laboratory of Neuromodulation, Beijing, China; ^3^Department of Neurology, Beijing Renhe Hospital, Beijing, China; ^4^Center of Epilepsy, Beijing Institute for Brain Disorders, Capital Medical University, Beijing, China

**Keywords:** sensorimotor integration, restless legs syndrome, transcranial magnetic stimulation, short latency afferent inhibition, long latency afferent inhibition

## Abstract

**Objective:** Restless legs syndrome (RLS) is a complicated sensorimotor syndrome that may be linked to changes in sensorimotor integration. The mechanism of such changes is unclear. The aim of this study was to investigate sensorimotor integration in patients with RLS through transcranial magnetic stimulation-motor evoked potentials (TMS-MEPs) preceded by peripheral electric stimulation.

**Methods:** Fourteen RLS patients and 12 healthy, age-matched controls were investigated. The clinical severity of RLS was evaluated based on the International Criteria of the International Restless Legs Syndrome Study Group (IRLSSG) severity scores. The tibial and median H-reflexes and the resting motor threshold (RMT) of the abductor pollicis brevis (APB) were tested in all 26 subjects. The RMT of the tibialis anterior (TA) was tested in 8 patients and 7 controls. All 26 subjects underwent measurement of unconditioned MEPs of the APB. Electric pulses were applied to the right median nerve, followed by TMS pulses over the left motor cortex at interstimulus intervals (ISIs) of 20, 25, 30, 50, 100, 150, and 200 ms. Unconditioned MEPs of the TA were measured in 8 patients and 7 controls. Electric pulses were applied to the right peroneal nerve, followed by TMS pulses over the left motor cortex at ISIs of 30, 35, 45, 60, 100, and 200 ms. The degree of modulation of MEPs by electric stimulation was expressed as the ratio of the conditioned MEP amplitude to the unconditioned MEP amplitude. Ratios <1 indicated inhibition, and ratios >1 indicated facilitation.

**Results:** No significant differences in RMT or H-reflex latencies or amplitudes were found between RLS patients and controls. A significant increase in unconditioned MEP amplitudes of the TA was observed in patients compared to controls (*p* = 0.03). Long-latency afferent inhibition (LAI) of the median nerve in RLS patients was decreased significantly at ISIs of 150 (*p* = 0.000) and 200 ms (*p* = 0.004). Upon peroneal nerve stimulation, no significant difference was observed between the two groups at any ISI.

**Conclusions:** Our results suggest increased motor cortical excitability of the legs and disturbed sensorimotor integration in RLS patients; this disturbance might originate at the cortical level.

## Introduction

Restless legs syndrome (RLS) is a complicated sensorimotor syndrome characterized by an urge to move the legs in association with unpleasant paresthesias (1). Various studies suggest that genetics, iron deficiency, disturbances in the dopaminergic system and abnormality in spinal conduction pathways are associated with the disorder (2–5). Although the pathology of RLS is increasingly understood, a clear physiological understanding remains elusive.

In most cases, the unpleasant sensation worsens at rest and at night and is usually relieved by movement; therefore, there appears to be a close relationship between sensory and motor systems in the pathology of RLS. Some imaging and electrophysiological studies have shown cerebral structural changes involving sensory and motor systems in RLS, and an impairment of sensorimotor integration processing at the cerebral level is assumed based on these studies (6–10). Sensorimotor integration is defined as a tight integration of the sensory and motor systems necessary for planning and carrying out precise movements. Abnormal sensorimotor integration may contribute to motor disorders, such as RLS. An efficient approach to study sensorimotor integration is to combine peripheral nerve stimulation with transcranial magnetic stimulation-motor evoked potentials (TMS-MEPs) to explore the interaction between input and output circuits at the cortex. There were several reported studies which used this approach. They only investigated upper limbs, and some of the results were inconsistent with each other. Rizzo et al. found short-latency afferent inhibition (SAI) significantly deficient in untreated RLS patients and reverted to normal after dopaminergic treatment (11); however, Bocquillon et al. did not find difference in SAI between patients and healthy controls (12). Compared with healthy controls, no difference of long-latency afferent inhibition (LAI) was found in Rizzo's study, while a lack of afferent-induced facilitation (AIF) was found in Bocquillon's study.

In the present study, we aimed to gain more insight into the underlying physiology of RLS by investigating sensorimotor integration in that disorder. Toward this goal, we studied the effect of median nerve and peroneal nerve stimulation on motor cortex excitability as indicated by MEPs, and we compared the effect between patients and healthy controls.

## Materials and methods

### Subjects

We included 14 patients with untreated idiopathic RLS (8 men and 6 women, median [Q1; Q3], age 61 [49; 64.5] years) diagnosed at the sleep clinic of our hospital by an experienced neurologist with sleep medicine expertise (YPW) according to the International Criteria of the International Restless Legs Syndrome Study Group (IRLSSG) confirmed in 2003 (13) and 12 healthy, age-matched controls (7 men and 5 women, median [Q1; Q3] age 49 [41.8; 57.8] years). All subjects who abused alcohol, caffeine or tea, and those with other neurological or metabolic diseases were excluded. The clinical severity of RLS was evaluated based on IRLSSG severity scores (13). All subjects were right-handed. Five patients had symptoms involving the arms in addition to the legs. None of the subjects was taking any dopamine agonists, psycholeptics, benzodiazepines or other medications that might influence cortical excitement. All subjects provided written informed consent for the study and publication. All procedures of the study had the approval of the Xuanwu Hospital Ethics Committee and were in accordance with the Declaration of Helsinki.

## Protocols

### Resting motor threshold (RMT)

All tests in this study were performed in the morning, from 9:00 AM to 11:00 AM. Three female patients and five female controls had menstrual cycles, and we performed evaluations at day 8 (±1 day) after the onset of menstruation. All 26 subjects had the RMT of the right abductor pollicis brevis (APB) measured. Eight patients and 7 controls also had the RMT of the right tibialis anterior (TA) measured. Each patient was seated in a comfortable chair, and TMS was performed with a Magstim system (Magstim Super Rapid Stimulator, Magstim Company, Whitland, Dyfed, UK) with a figure-eight coil. The coil was placed tangentially to the scalp with the handle pointing backwards and laterally at a 45° angle from the midline. The optimal stimulation spot was located by stimulating the presumed primary motor cortex every 1 cm in distance in a 6 cm square contralateral to the more symptomatic side, and the identified spot was marked on the scalp to ensure identical placement of the coil throughout the experiment. The optimal stimulation spot for the APB was usually located 2 cm anterior and 2 cm lateral to Cz (referring to the international standardized 10–20 system of electrode placement). The optimal stimulation spot for the TA was usually located near Cz. The surface EMG was recorded with disc-shaped Ag-AgCl electrodes placed in a tendon-belly arrangement. RMT was defined as the minimal magnetic stimulus intensity that produced a TMS-MEP of peak-to-peak amplitude >50 mV in the relaxed APB or TA muscle in 5 of 10 trials. The signal was amplified, filtered (bandpass 20 Hz−3 kHz, gain 100 μV/Div for the APB and 50 μV/Div for the TA), displayed and stored in a laboratory computer for off-line analysis. All subjects relaxed throughout the study. Trials contaminated with voluntary muscle activity were rejected.

### H-reflex

All 26 subjects were tested. Electric stimuli were delivered and compound action potentials recorded by a da Vinci physiological response recorder.

In the tibial H-reflex test, the subject rested in the prone position on a bed. The H-reflex was measured by stimulating (duration 0.2 ms, frequency 0.2 Hz, bandpass 20 Hz to 10 kHz, gain 5 μV/Div) the right tibial nerve 10 times in the popliteal fossa with the anode distal and the cathode proximal (2 cm apart) and recording the responses from Ag-AgCl surface electrodes on the soleus muscle. The current intensity was increased gradually and adjusted to the intensity with the shortest latency. In the median H-reflex test, the subject was seated comfortably in a reclining armchair. The APB was kept in isometric contraction to obtain a 20% maximum amplitude. The median H-reflex was measured by stimulating (duration 0.2 ms, frequency 1 Hz, bandpass 20 Hz to 10 kHz, gain 5 μV/Div) the median nerve 10 times at the right wrist with the anode distal and the cathode proximal (2 cm apart) and recording the responses from Ag-AgCl surface electrodes on the APB. The current intensity was adjusted to evoke a slight thumb twitch. Ten latencies and amplitude values of the H-reflex were measured and averaged.

### Unconditioned MEP

Unconditioned MEPs of the right APB were measured in all 26 subjects. Unconditioned MEPs of the right TA were measured in 8 patients and 7 controls. The intensity of the magnetic test stimulus administered to the contralateral motor cortex was 120% of the RMT. Eight amplitude values of unconditioned MEPs were measured and averaged.

### TMS-MEP conditioning with median nerve stimulation

All 26 subjects were tested. Electric stimuli were delivered by a Grass S88 stimulator. The conditioning stimuli were single electric pulses (duration 0.2 ms) applied to the median nerve at the wrist through electrodes, with the cathode positioned proximally. The current intensity was adjusted to evoke a slight thumb twitch. The intensity of the magnetic test stimulus given to the contralateral motor cortex was 120% of the RMT. The conditioning stimulus to the median nerve preceded the magnetic test stimulus with 20, 25, 30, 50, 100, 150, and 200 ms ISIs. Eight stimuli at each ISI were delivered in a pseudorandom order.

The modulation degree of MEP by median nerve stimulation was expressed as the ratio of the conditioned MEP amplitude to the unconditioned MEP amplitude. Ratios <1 represented inhibition, and ratios >1 represented facilitation.

### TMS-MEP conditioning with peroneal nerve stimulation

Eight patients and 7 controls were tested. Electric stimuli were delivered by a Grass S88 stimulator. The conditioning stimuli were single electric pulses (duration 0.2 ms) applied to the peroneal nerve at the fibulae capitulum through electrodes with the cathode positioned proximally. The current intensity was adjusted to evoke a slight foot dorsiflexion. The intensity of the magnetic test stimulus given to the contralateral motor cortex was 120% of the RMT. The conditioning stimulus to the peroneal nerve preceded the magnetic test stimulus with 30, 35, 45, 60, 100, and 200 ms ISIs. Eight stimuli at each ISI were delivered in a pseudorandom order.

The degree of MEP modulation by peroneal nerve stimulation was expressed as the ratio of the conditioned MEP amplitude to the unconditioned MEP amplitude. Ratios <1 represented inhibition, and ratios >1 represented facilitation.

### Statistical analysis

All analyses were conducted using SPSS 13 (SPSS Inc., Chicago, IL). *T*-Test was used to compare the ages between groups. One-way analysis of variance was used to compare the latency and amplitude values of the H-reflex, the RMT and the amplitude values of the unconditioned MEPs between groups. Repeated-measures analysis of variance was used to compare the ratios of conditioned to unconditioned MEP amplitude between groups at different ISIs. If the group effect was significant, Fisher's protected least significant difference post hoc test was conducted. All the data are shown as the mean ± standard error of the mean unless otherwise stated. *P*-values < 0.05 were considered statistically significant.

## Results

No adverse side effects were reported during the study.

### Clinical assessment of RLS

For the study of median nerve stimulation, in all 26 subjects enrolled, there was no significant difference in age between patients and controls (*p* = 0.194). For the study of peroneal nerve stimulation, in all 15 subjects enrolled, there was no significant difference in age between patients and controls (*p* = 0.522).

In the patient group, the median [Q1; Q3] IRLSSG severity score was 26.5 [17.3; 33.8]. The median [Q1; Q3] age of onset was 45.5 [33; 56] years.

### RMT

**Figure 1:**
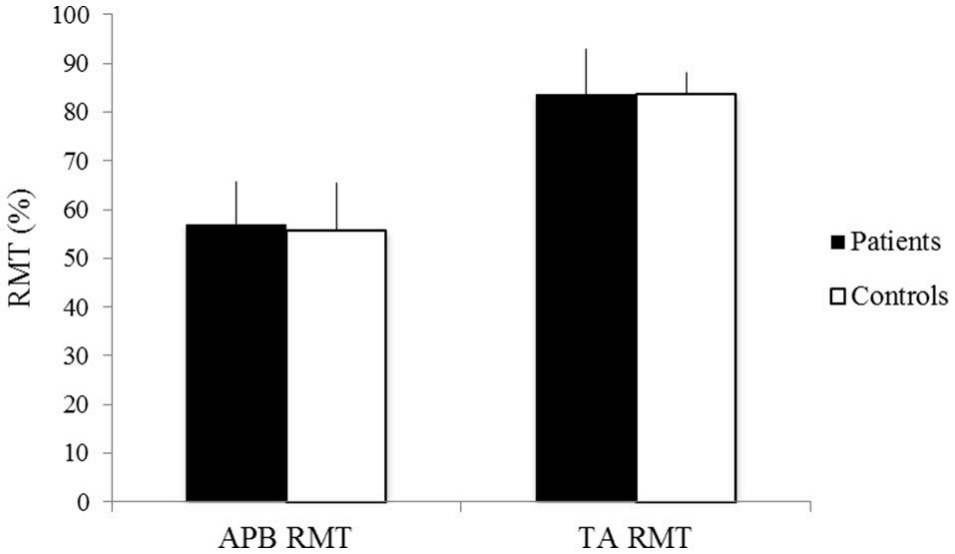
RMT of the APB and TA of patients (black) and controls (white). X-axis: RMT of the APB (left) and the TA (right). Y-axis: RMT (%). Vertical lines are standard errors.

There was no significant difference in the RMT of the APB between patients and controls (patients 57.1 ± 8.6%, controls 55.8 ± 9.7%, *p* > 0.05) (Figure 1). The RMT of the TA also did not differ between groups (patients 83.6 ± 9.3%, controls 83.6 ± 4.5%, *p* > 0.05) (Figure 1).

### H-reflex tests

**Figure 2:**
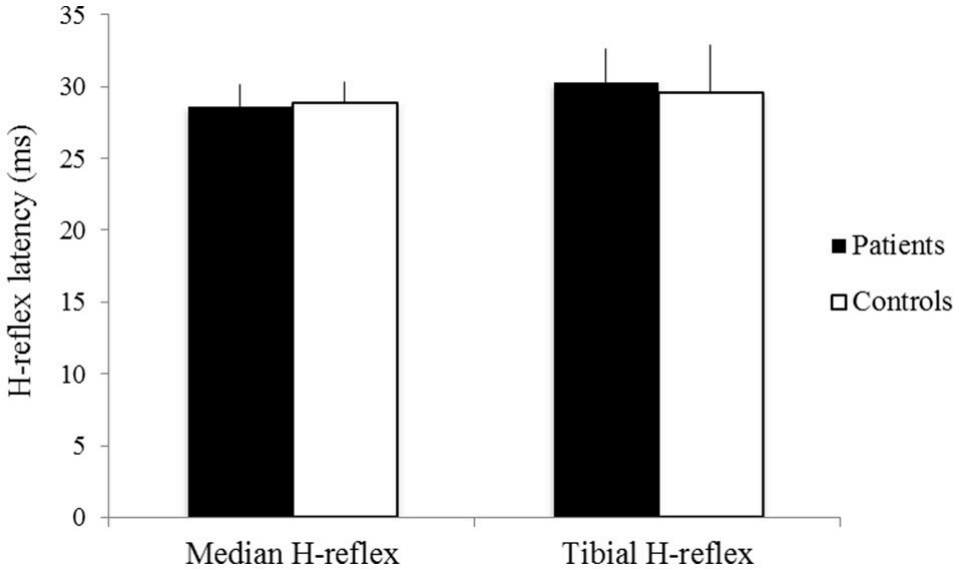
The latencies of the median H-reflex and tibial H-reflex of patients (black) and controls (white). X-axis: median H-reflex (left) and tibial H-reflex (right). Y-axis: latency (ms). Vertical lines are standard errors.

**Figure 3:**
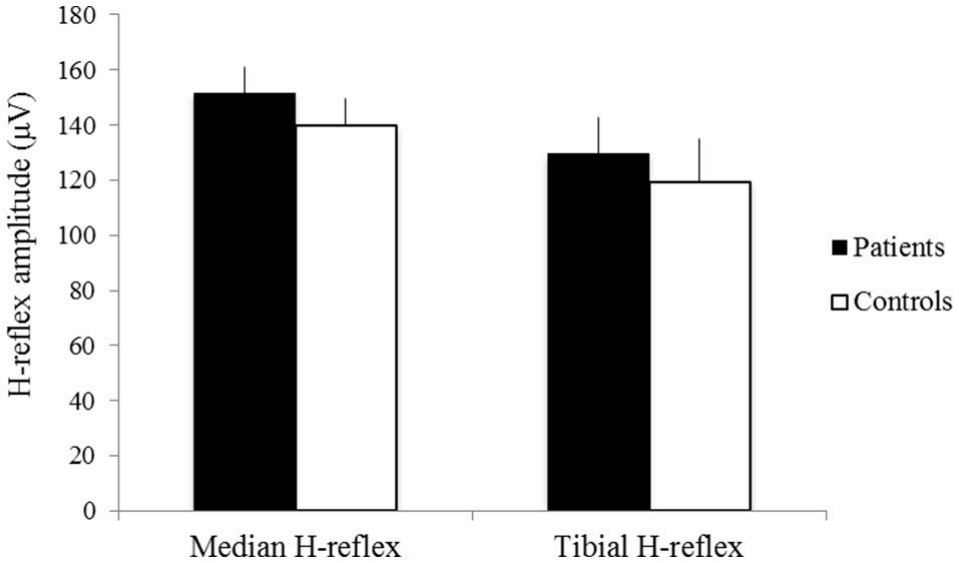
The amplitudes of the median H-reflex and tibial H-reflex of patients (black) and controls (white). X-axis: median H-reflex (left) and tibial H-reflex (right). Y-axis: amplitude (μV). Vertical lines are standard errors.

The latencies of the median H-reflex and tibial H-reflex showed no significant difference between groups (patients: mean latency of the median H-reflex 28.62 ± 1.56 ms, mean latency of the tibial H-reflex 30.32 ± 2.28 ms; controls: mean latency of the median H-reflex 28.89 ± 1.41 ms, mean latency of the tibial H-reflex 29.60 ± 3.27 ms *p* > 0.05) (Figure 2). The amplitudes of the median H-reflex and tibial H-reflex also did not show significant differences between patients and controls (patients: mean amplitude of the median H-reflex 151.58 ± 9.52 μV, mean amplitude of the tibial H-reflex 129.62 ± 13.10 μV; controls: mean amplitude of the median H-reflex 139.63 ± 10.01 μV, mean amplitude of the tibial H-reflex 119.25 ± 15.63 μV, *p* > 0.05) (Figure 3).

### Unconditioned MEPs

**Figure 4:**
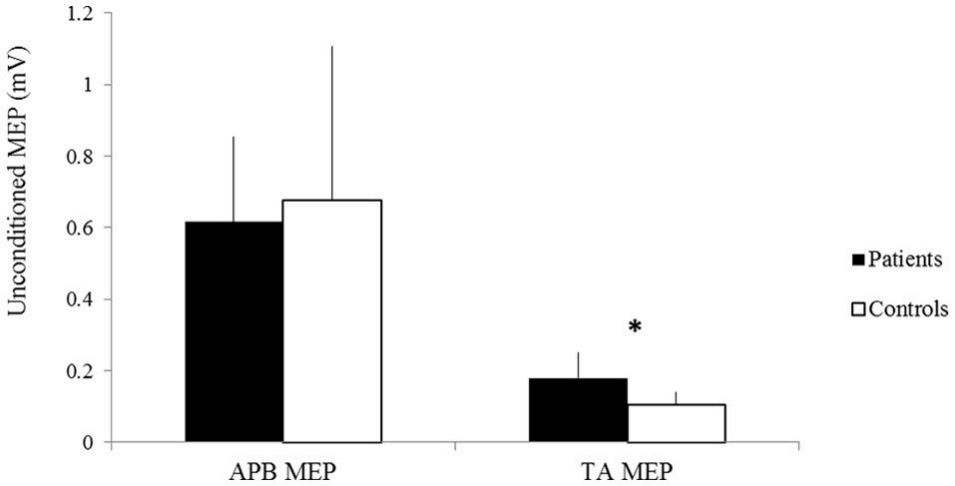
The amplitudes of unconditioned MEPs of the APB and TA of patients (black) and controls (white). X-axis: unconditioned MEPs of the APB (left) and the TA (right). Y-axis: amplitude of unconditioned MEPs (mV). Vertical lines are standard errors. *p < 0.05.

No significant difference was observed in the amplitude of unconditioned MEPs of the APB between patients and controls (patients 0.62 ± 0.24 mV, controls 0.68 ± 0.43 mV, *p* > 0.05) (Figure 4). The amplitude of unconditioned MEPs of the TA significantly differed between groups (patients 0.18 ± 0.07, controls 0.11 ± 0.03, *p* = 0.03) (Figure 4).

### Recovery curve of MEP conditioning with median nerve stimulation

**Figure 5:**
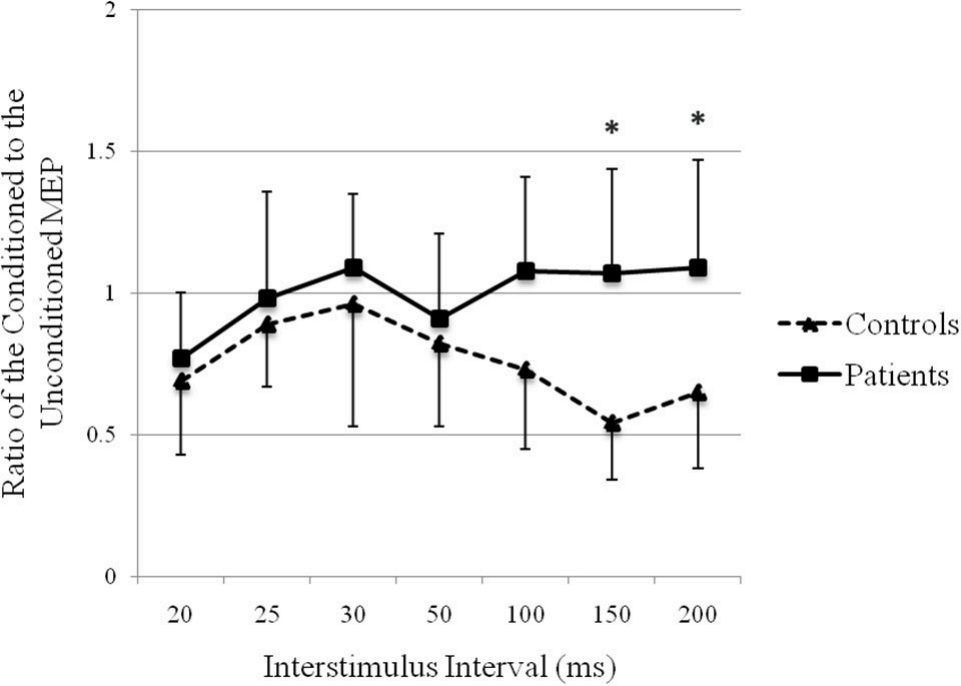
Recovery curve of MEP conditioning with median nerve stimulation. X-axis: ISI between peripheral nerve conditioning stimulus and magnetic stimulus. Y-axis: ratio of the conditioned to the unconditioned MEP amplitude. Vertical lines are standard errors. *p < 0.05.

The recovery curve of MEP conditioning with median nerve stimulation is shown in Figure 5. There was inhibition at all ISIs in controls and at ISIs of 20, 25, and 50 ms in patients. There was facilitation at ISIs of 30, 100, 150, and 200 ms in patients. There was a significant difference in the ratios of the conditioned to the unconditioned MEP amplitude between groups (repeated-measures analysis of variance: *F*_(1, 24)_ = 7.45, *p* < 0.05). The post hoc test showed significant facilitation at ISIs of 150 ms (*p* = 0.000) and 200 ms (*p* = 0.004) in patients compared with that in controls.

### Recovery curve of MEP conditioning with peroneal nerve stimulation

**Figure 6:**
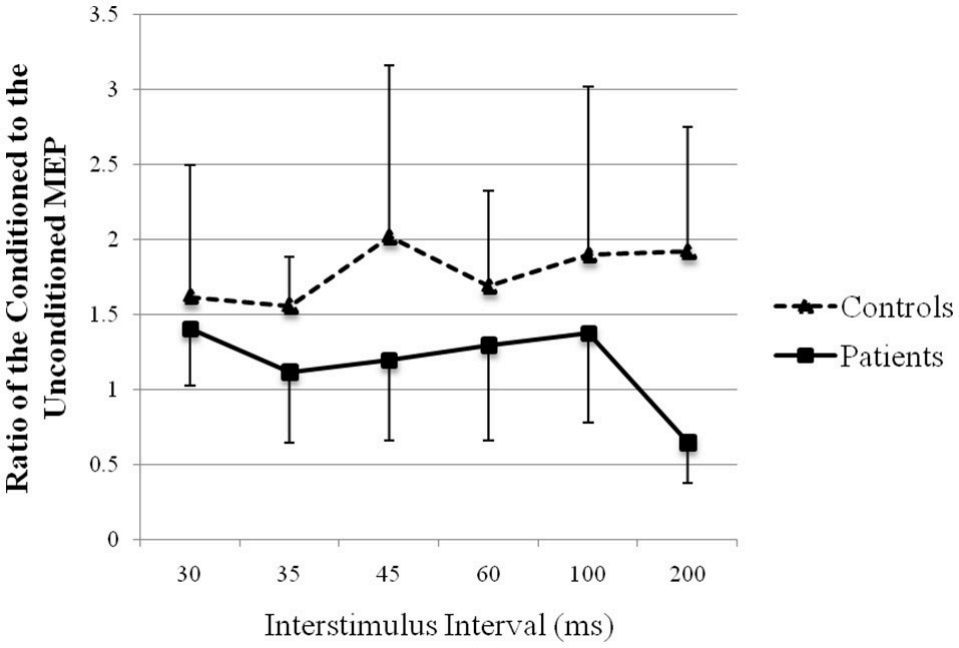
Recovery curve of MEP conditioning with peroneal nerve stimulation. X-axis: ISI between peripheral nerve conditioning stimulus and magnetic stimulus. Y-axis: ratio of the conditioned to the unconditioned MEP amplitude. Vertical lines are standard errors.

The recovery curve of MEP conditioning with peroneal nerve stimulation is shown in Figure 6. There was facilitation at all ISIs in both groups, except 200 ms in patients. Although the test response was facilitated more strongly in controls than that in patients, it showed no significant difference (repeated-measures analysis of variance: *F*_(1, 13)_ = 1.75, *p* > 0.05).

## Discussion

### Motor cortical excitability

Our study found no significant difference in RMTs between patients and controls. This finding is consistent with those of previous studies (14, 15). A significant increase in unconditioned MEPs was found in the TA but not in the APB in patients compared to controls, which probably indicates an increase in motor cortical excitability of the legs in RLS patients at rest. Previous studies have reported similar findings of hand MEPs in RLS patients. Studies examining MEPs in the TA are relatively rare. Quatrale et al. (16) and Tergau et al. (9) assessed MEPs in the TA and did not find any significant difference compared to controls. The reason for this inconsistency is unclear, but the preactivation hypothesis might play a role in this discrepancy. The MEP size is highly influenced by the presence of background muscle activity (17). Although all measurements were performed in the morning and patients were relaxed during the tests, a subliminal preactivation of muscles could not be excluded in our study.

### Spinal excitability

Currently, the spinal cord is thought to be included in RLS pathogenesis (18). Our study found no significant difference in H-reflex latencies or amplitudes between patients and controls. This result was consistent with Scaglione's study (19), which enrolled 7 patients and 10 healthy controls and found that the two groups did not differ in H-reflex mean threshold, latency or the Hmax/Mmax ratio. However, they found a significant reduction in Ib inhibition of H-reflex conditioning ISI, which indicated altered group I nonreciprocal inhibition enhances the spinal circuit excitability of RLS patients. Therefore, the H-reflex latency and amplitude results in this population cannot exclude spinal involvement in RLS. Additional tests are needed to further investigate spinal excitability.

### Sensorimotor integration

There is a complicated interaction between the motor and sensory system in the brain. Sensory input is integrated to assist in the execution of motor programs. The excitability of the motor cortex can be modulated by peripheral sensory stimulation, which reflects the interaction between input and output circuits at the cortical level. The combination of peripheral nerve stimulation with TMS-MEP provides an efficient approach to study sensorimotor integration. This modulatory effect depends on the ISI between the conditioning electric stimulus and the magnetic stimulus. An inhibitory effect, known as SAI, occurs at ISIs as short as 20 ms (20, 21). At ISIs longer than 100 ms, there is a similar effect known as LAI (22, 23). At ISIs between 45 and 60 ms, AIF is observed (12). Our study showed significantly reduced LAI of hand motor cortical excitability in RLS patients compared with that in healthy controls. SAI and LAI are mediated through different sensorimotor circuits. SAI, with a short stimulation interval, is considered to reflect the direct inhibitory effect of sensory input on motor output, which requires only a few processing steps (21, 23, 24). LAI, with a longer stimulation interval, involves more sensory processing structures including the primary somatosensory area, secondary somatosensory area and basal ganglia-thalamocortical loop (25). Therefore, our results suggested that the pathophysiology of RLS might be based on abnormal sensory processing involving these structures or pathways rather than direct sensorimotor connections at the cortex. This impairment of sensorimotor integration was found in LAI of the upper limbs, even though the clinical symptoms of the patients were mainly in the lower limbs. We did not observe significantly reduced SAI in RLS patients. However, a trend of SAI reduction was shown in our study. The issue of SAI in RLS was controversial in two previously reported studies (11, 12). Rizzo et al. found that SAI was significantly deficient in RLS patients and reverted to normal after dopaminergic treatment; however, Bocquillon et al. found no difference in SAI between patients and healthy controls.

RLS is considered a complicated sensorimotor syndrome rather than a purely motor disease. Previous studies have shown structural sensorimotor gray matter alterations in RLS patients (6, 7). Etgen et al. (6) found a significant bilateral gray matter increase in the pulvinar in RLS patients compared to controls. These changes in thalamic structures probably resulted from a chronic increase in afferent input of behaviorally relevant information. Unrath (7) and Chang (26) found a significant regional decrease of gray matter volume in the primary somatosensory cortex in RLS patients. These results provide anatomical evidence of sensorimotor gray matter alterations relevant to RLS. Based on these findings, as well as the results of our study, we speculate that a disturbance of sensorimotor integration at the cerebral level may contribute to RLS. Sensory input may participate in the release of untoward motor sequences and lead to involuntary movements (11).

### Differences in findings for the upper and lower limbs

The most impressive motor phenomena of RLS characteristically involve the lower limbs. The efficacy of dopamine D2/D3 receptor agonists in the treatment of RLS suggests an important role of dopaminergic dysfunction in the pathogenesis of the disorder (27). Levant et al. examining slide-mounted tissue sections from healthy rats by quantitative autoradiography, found the highest densities of D3 dopamine receptors in the superficial layers of the dorsal horn at the lumbar levels (28). This finding supports the common involvement of the legs in RLS patients. Nevertheless, RLS can involve not only the legs but also the arms. In our study, all patients had notable leg symptoms, and 5 of them had arm symptoms. We tested the effect of median nerve stimulation in all subjects, but owing to the difficulty of obtaining TA MEPs, the effect of peroneal nerve stimulation was measured in only 8 patients and 7 controls. The responses to peroneal nerve stimulation were facilitated in both patients and controls. Unexpectedly, there was less facilitation of peroneal nerve stimulation in patients than that in controls, although not significantly less.

The mechanism of this novel finding is unclear. Another study of TMS-MEP conditioning with peripheral inputs in RLS showed a similar lack of AIF in patients compared with that in healthy controls (12), but this result was observed in the upper limbs. This finding of reduced facilitation was consistent with our previous functional MRI study, which showed that activity in sensorimotor regions was decreased in RLS and elevated after treatment (29). Because all patients had clinical symptoms affecting the legs, we speculated that a compensation in the leg area of the primary motor cortex might develop in these 8 patients, owing to a greater disturbance of sensorimotor integration. In contrast, no compensation was observed in the arm area in any of the 14 patients, probably due to the lesser involvement of the arms.

There is also another possible explanation for the reduced facilitation of peroneal nerve stimulation in patients. It could be assumed that a subliminal preactivation of the lower limb muscles is present in RLS patients. In an activated muscle, intracortical inhibition (ICI) and intracortical facilitation (ICF) in cortical areas that project to the active muscle are both reduced compared to the resting state muscle (30). This is supported by the findings in Tergau's study, which showed that ICF was increased in the abductor digiti minimi (ADM) but reduced in the clinically affected abductor hallucis muscle (AH), and the patients with more severe disease showed more flattening of the recovery curve (9). In our study, although all measurements were performed under resting conditions and in the morning, this subclinical preactivation might weaken cortical facilitation.

Additionally, inhibitory but not facilitatory circuits of upper and lower limb motor cortex are interconnected (31). Therefore, the differences in findings for the upper and lower limbs in RLS patients could be related to differences in the involvement of facilitatory and inhibitory circuits of the leg compared to the hand motor cortex (16).

There are few reported studies of sensorimotor integration in RLS, and only the upper limbs were studied (11, 12). To the best of our knowledge, the present study is the first to explore the sensorimotor integration of the lower limbs in RLS. This study found a significant alteration in cortical sensorimotor integration of the upper limbs and potential impairment of the lower limbs. There were several limitations of the study. First, this study had a limited sample size. Second, multiple statistical tests in our study might raise the risk of false positives. For example, the *p*-value (0.03) of unconditioned MEPs of the TA might be insignificant if adjusted for multiple analysis. We did not adjust for multiple analysis because this was a preliminary exploration. Further large sample-size studies were warranted to confirm our results. Additional investigations, such as studies based on a combination of comprehensive neurophysiologic techniques and functional imaging, are warranted to clarify the neurotransmitters and pathways involved in impaired sensorimotor integration in RLS.

## Conclusion

In conclusion, this study suggested that the motor cortical excitability of the legs was increased and that the cortical motor output in response to sensory input in LAI was altered in RLS, indicating impaired sensorimotor integration at the cortical level. This finding supports a potential association between sensorimotor integration and sensory symptoms in RLS, although further confirmation is warranted in larger sample sizes, and the mechanism needs to be characterized accurately in multiple modalities.

## Author contributions

YL, YiW, and YuW contributed at all stages of manuscript preparation. YL wrote the manuscript. All authors were involved in data recording and discussed the results.

### Conflict of interest statement

The authors declare that the research was conducted in the absence of any commercial or financial relationships that could be construed as a potential conflict of interest.
